# Assessment of Tree-Based Statistical Learning to Estimate Optimal Personalized Treatment Decision Rules for Traumatic Finger Amputations

**DOI:** 10.1001/jamanetworkopen.2019.21626

**Published:** 2020-02-21

**Authors:** Kelly A. Speth, Alfred P. Yoon, Lu Wang, Kevin C. Chung

**Affiliations:** 1 Department of Biostatistics, University of Michigan School of Public Health, Ann Arbor; 2 Section of Plastic Surgery, Michigan Medicine, Ann Arbor; 3 Section of Plastic Surgery, Department of Surgery, University of Michigan Medical School, Ann Arbor

## Abstract

**Importance:**

Optimal treatment for traumatic finger amputation is unknown to date.

**Objective:**

To use statistical learning methods to estimate evidence-based treatment assignment rules to enhance long-term functional and patient-reported outcomes in patients after traumatic amputation of fingers distal to the metacarpophalangeal joint.

**Design, Setting, and Participants:**

This decision analytical model used data from a retrospective cohort study of 338 consenting adult patients who underwent revision amputation or replantation at 19 centers in the United States and Asia from August 1, 2016, to April 12, 2018. Of those, data on 185 patients were included in the primary analysis.

**Exposures:**

Treatment with revision amputation or replantation.

**Main Outcomes and Measures:**

Outcome measures were hand strength, dexterity, hand-related quality of life, and pain. A tree-based statistical learning method was used to derive clinical decision rules for treatment of traumatic finger amputation.

**Results:**

Among 185 study participants (mean [SD] age, 45 [16] years; 156 [84%] male), the median number of fingers amputated per patient was 1 (range, 1-5); 115 amputations (62%) were distal to the proximal interphalangeal joint, and 110 (60%) affected the nondominant hand. On the basis of the tree-based statistical learning estimates, to maximize hand dexterity or to minimize patient-reported pain, replantation was found to be the best strategy. To maximize hand strength, revision amputation was the best strategy for patients with a single-finger amputation but replantation was preferred for all other injury patterns. To maximize patient-reported quality of life, revision amputation was the best approach for patients with dominant hand injuries, and replantation was the best strategy for patients with nondominant hand injuries.

**Conclusions and Relevance:**

The findings suggest that the approach to treating traumatic finger amputations varies based on the patient’s injury characteristics and functional needs.

## Key Points

**Question:**

How can patient heterogeneity be accommodated and patients’ individual information used to guide treatment selection for traumatic finger amputation to improve long-term outcomes?

**Findings:**

This decision analytical model of 185 patients requiring traumatic finger amputation estimated that to maximize hand strength, replantation was the preferred approach for older patients and those with multifinger amputations; to maximize quality of life, revision amputation was the preferred approach for patients with dominant hand injuries; and to maximize dexterity and minimize pain, replantation was the preferred approach.

**Meaning:**

The findings suggest that treatment approaches for traumatic finger amputation differ based on the patient’s injury characteristics and functional needs.

## Teaser

This decision analytical model used tree-based statistical learning to estimate evidence-based treatment assignment rules to enhance long-term functional and patient-reported outcomes in patients after traumatic finger amputation.

## Introduction

Traumatic finger amputations affect more than 45 000 people a year in the United States.^1,2^ Recovery after amputation includes rebuilding manual strength, flexibility, and sensation as well as managing recurrent pain and psychosocial consequences that result from the absence or deformity of fingers.^3,4,5,6^ The decision for surgical treatment for traumatic finger amputation is made on a case-by-case basis and typically includes replantation or primary closure of the amputated stump (revision amputation). Although there are de facto indications that guide the surgeon’s decision of replantation or revision amputation,^7^ to date, there are no formal clinical practice guidelines available to the surgeon.

Personalized medicine and evidence-based care are driving factors behind today’s clinical care. Because traumatic finger amputation is unexpected and the patient’s presentation to the emergency department requires rapid triaging and decision-making by the physician, the objective of our analysis was to establish evidence-based, personalized treatment assignment rules to assist surgeons in selecting treatment that will optimize long-term patient-reported outcomes and function. Whereas there has been an abundance of research to address this question,^6,7,8,9,10,11,12,13^ this analysis used broad, composite outcomes and a tree-based reinforcement learning method based on concepts from causal inference^14^ and machine learning.^15^ Applying data collected from the Finger Replantation and Amputation Challenges in Assessing Impairment, Satisfaction, and Effectiveness (FRANCHISE) study,^10^ which was, to our knowledge, the largest multicenter retrospective cohort study of long-term outcomes in patients with traumatic finger amputation treated with replantation or revision amputation, we implemented a tree-based reinforcement learning method to derive a clinical decision rule for each of 4 broad categories of hand outcomes: hand strength, dexterity, pain, and patient-reported quality of life (QOL). We sought to develop and test the feasibility of a decision support tool that could assist surgeons in making personalized treatment decisions for patients with traumatic finger amputation and to contribute to the body of literature considered when developing or updating clinical practice guidelines.

## Methods

### Study Population

**Figure 1.**
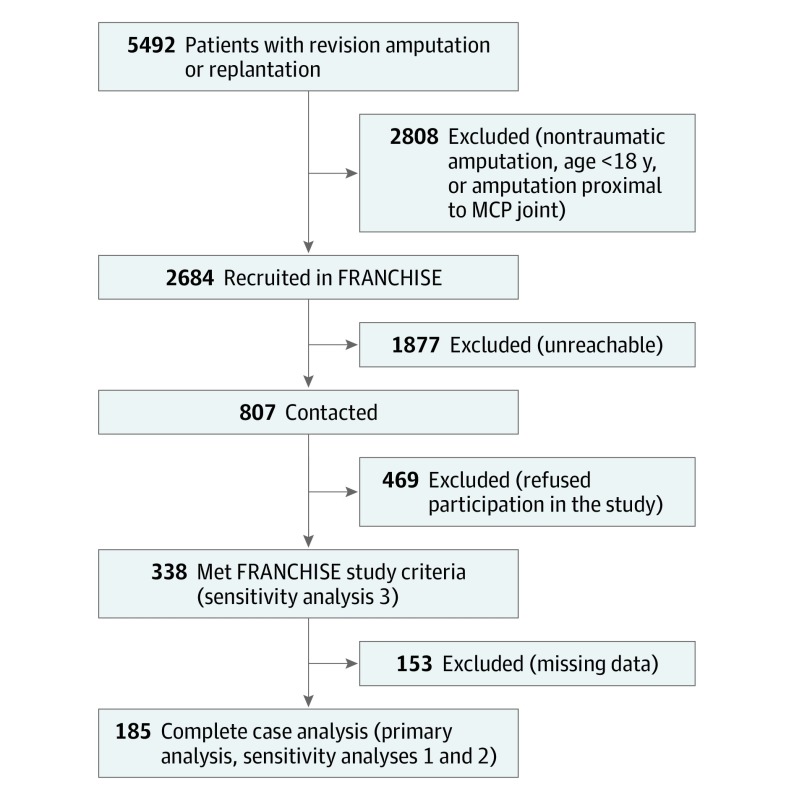
Screening and Enrollment for the FRANCHISE (Finger Replantation and Amputation Challenges in Assessing Impairment, Satisfaction, and Effectiveness) Study MCP indicates metacarpophalangeal.

This decision analytical model was conducted using data from the FRANCHISE study,^10^ which include baseline characteristics and patient-reported and functional outcome measures for 338 consenting adults with traumatic amputation of fingers distal to the metacarpophalangeal joint who were treated by revision amputation or successful replantation at least 1 year before recruitment. Of those, 185 patients were included for complete case analysis (Figure 1). The retrospective cohort study was conducted at 19 multinational sites in the United States and Asia between August 1, 2016, and April 12, 2018. The research protocol was approved by the participating sites’ local institutional review boards. The current study was approved by the University of Michigan institutional review board. All participants gave written informed consent in their native language, and all data were deidentified. Additional details on the original study design and enrollment and a comprehensive description of collection methods for functional assessments and patient-reported outcomes are found in the article by Chung et al.^10^ This study followed the Strengthening the Reporting of Observational Studies in Epidemiology (STROBE) reporting guideline.

### Outcomes

Four outcome measures were considered in this analysis: hand strength, dexterity, pain, and patient-reported hand QOL. The strength outcome was a composite derived using measures of grip strength, lateral pinch strength, 2-point pinch strength, and 3-point pinch strength for the healthy and injured hands. We standardized the difference between the injured and healthy hands and computed the mean across all measures. The dexterity outcome was developed from the timed 9-hole test as the standardized difference between the healthy and injured hands. The pain outcome was the standardized difference in self-reported pain between the healthy and injured hands (difference calculated as pain-healthy – pain-injured, in which both were collected using a scale of 0-100, with larger values representing more pain), assuming that pain in the healthy hand was less than or equal to that of the injured hand. For each of the aforementioned outcomes, larger values of the standardized differences between the injured and healthy hands were preferred because those demonstrated a recovery to at or near that of the healthy hand. In addition, patient-reported hand QOL was derived from the Michigan Hand Outcomes Questionnaire^16,17^ (MHQ) and Disabilities of the Arm, Shoulder, and Hand^18,19^ (DASH) scores. Because higher MHQ and lower DASH scores represent improved QOL, DASH scores were reversed (calculated as 100 − DASH) to align preferences toward a higher score. The QOL outcome was taken as the mean of the standardized MHQ and reverse DASH outcomes.

### Tree-Based Reinforcement Learning

**Figure 2.**
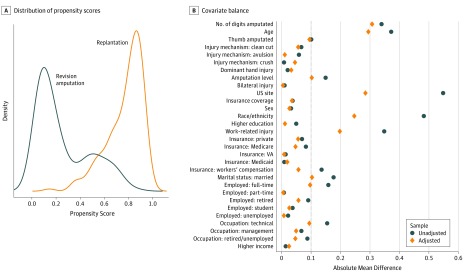
Propensity Score Diagnostics Including Distribution and Covariate Balance A, Distributions of the estimated propensity scores for each patient are plotted by the patient’s actual treatment assignment. B, Covariate balance across patient demographic and injury characteristics was assessed before and after propensity score adjustment using absolute mean differences. A value of 0.1 or less is generally considered to be adequately balanced. We observed balance across most covariates, although adjusted absolute mean differences for number of fingers amputated, age, US site, race/ethnicity, and work-related injury demonstrated poor covariate balance. There was a high degree of pairwise correlation between the variables US site, race/ethnicity, and work-related injury (all *P* < .001).

On the basis of the work of Tao et al,^20^ we used a tree-based reinforcement learning (T-RL) method to conduct a complete case analysis. The T-RL method is a semiparametric statistical learning method that can be applied to observational data with the goal of elucidating a personalized treatment decision rule that uses the individual-level tailoring variables to estimate a long-term counterfactual outcome. It uses a modification of a standard regression tree to reflect that the target of estimation, that is, a treatment rule rather than a continuous outcome, is not directly observed. Unlike standard regression trees that often perform covariate splits using the sum of squared prediction errors,^15^ the T-RL method uses a splitting criterion (ie, a purity measure) defined by the expected counterfactual mean outcome, a concept that incorporates the propensity score to adjust for baseline covariate imbalances in observational data to address model misspecification. We estimated the propensity score using SuperLearner, an algorithm that uses cross-validation to estimate best fit across multiple machine learning models and then combines these predictions (ie, an ensemble method) using a weighted mean based on the test data performance.^21^ We made no a priori selection of covariates to include in the model, as suggested by Austin.^22^ The overlap of the propensity estimates based on the actual treatment received and covariate balance after propensity score adjustment is shown in Figure 2. Next, similar to conventional regression tree analysis, we specified a maximum tree depth of 2 and a minimum improvement in the purity measure of 5% needed for a new split. Additional details pertaining to the T-RL method are provided in the eAppendix in the Supplement.

### Explanatory Variables

Baseline covariates included patient factors and injury characteristics. Variables considered as candidates for a treatment decision rule (Table 1) included age, number of fingers amputated, thumb amputation, dominant hand injury, amputation level, mechanism of injury, and bilateral injury because these factors are clinically relevant to decision-making.^7,9,12,13,23,24^ In addition to these candidate variables, possible confounding variables included in the propensity model included sex, race/ethnicity, educational level, income level, marital status, employment status, occupation group, location of care, work-related injury, insurance, and insurance type.

**Table 1.** Candidate Covariates Included in Estimated Treatment Decision Rules for All Analyses

Analysis	Cohort	Candidate Covariates
Primary	Complete case (n = 185)	No. of fingers amputated (continuous), thumb amputated (binary), dominant hand injured (binary), mechanism of injury (nominal), amputation level (ordinal), bilateral injury (binary), and age (continuous)
Sensitivity analysis 1	Complete case (n = 185)	Injury group, as defined in Chung et al^10^: single finger (excluding thumb) amputation distal to the PIP joint; single finger (excluding thumb) amputation proximal to the PIP joint; thumb-only amputation distal to the IP joint; thumb-only amputation proximal to the IP joint; fingers amputated (excluding thumb); ≥3 fingers amputated (excluding thumb); 2 fingers amputated (including thumb); and ≥3 fingers amputated (including thumb)
Sensitivity analysis 2	Complete case (n = 185)	Same as primary analysis excluding age
Sensitivity analysis 3	Imputed (n = 338)	Same as primary analysis

Abbreviations: IP, interphalangeal; PIP, proximal interphalangeal.

### Sensitivity Analyses

We conducted sensitivity analyses to evaluate different sets of assumptions. First, we analyzed our cohort using just a single variable—an ordinal covariate that represented increasing degree of amputation severity—as a possible tailoring variable in the decision rule (sensitivity analysis 1) (Table 1 and Table 2); this analysis allowed for a comparison with results from the primary FRANCHISE analysis.^10^ Second, we repeated the first sensitivity analysis, but instead of an ordinal variable that summarized severity based on injury characteristics, we included all individual injury characteristics except age as possible variables in a decision rule (sensitivity analysis 2) (Table 1 and Table 2). This second setting also enabled us to compare the findings with the results of Chung et al^10^ and allowed the individual association of each injury characteristic with a decision rule to be assessed. Third, we performed the analysis using the full patient cohort, with missing covariate and outcome data imputed using the random forest method, which enabled us to demonstrate the utility of this method with nearly twice the sample size of the complete case analysis (sensitivity analysis 3) (Table 1 and Table 3). A comparison of demographic characteristics, injury characteristics, and outcome measures for the full imputed data set used in the third sensitivity analysis and the analysis data set is provided in the eTable in the Supplement.

**Table 2.** Estimated Decision Rules for All Optimization Goals and Analyses

Optimization Goal	Estimated Decision Rule			
	Primary Analysis (n = 185)	Sensitivity Analysis 1 (n = 185)	Sensitivity Analysis 2 (n = 185)	Sensitivity Analysis 3 (n = 338)
Hand function				
Strength	If the patient had 1 finger amputated and is aged ≤42 y, treat with revision amputation; otherwise, replantation	If the patient had 1 finger amputated proximal to the proximal interphalangeal joint or had a thumb amputated, treat with revision amputation; otherwise, replantation	If the patient had a thumb amputated (but no other fingers), treat with revision amputation; otherwise, replantation	If the patient had ≤3 fingers amputated and is aged ≤73 y, treat with revision amputation; otherwise, replantation
Dexterity	Treat all patients with replantation	Treat all patients with replantation	Treat all patients with replantation	If the patient is aged ≤58 y, treat with replantation; otherwise, revision amputation
Patient-reported				
Hand function	If the patient injured the dominant hand, treat with revision amputation; otherwise, replantation	Treat all patients with replantation	If the patient injured the dominant hand, treat with revision amputation; otherwise, replantation	If the patient injured the dominant hand, treat with revision amputation; otherwise, replantation
Pain	Treat all patients with replantation	Treat all patients with replantation	Treat all patients with replantation	If the patient is aged ≤37 y, treat with revision amputation; otherwise, replantation

**Table 3.** Summary Statistics for Patient Demographic Characteristics, Injury Characteristics, and Outcome Measures in the Analysis Cohort^a^

Variable	Overall (N = 185)	Revision Amputation (n = 54)	Replantation (n = 131)	*P* Value^b^
Demographic characteristic				
Age, mean (SD) [range], y	45 (16.0) [18-82]	49 (17.9) [21-82]	44 (15.0) [18-80]	.09
Sex				
Male	156 (84)	45 (83)	111 (85)	.99
Female	29 (16)	9 (17)	20 (15)	
Treatment location				
United States	27 (15)	15 (28)	12 (9)	.002
Asia	158 (85)	39 (72)	119 (91)	
Baseline injury characteristics				
Fingers amputated, median (IQR) [range], No.	1.0 (1-2) [1-5]	1.0 (1-2) [1-4]	1.0 (1-2) [1-5]	.01
Thumb amputated				
Yes	47 (25)	10 (18)	37 (28)	.23
No	138 (75)	44 (82)	94 (72)	
Mechanism of injury				
Clean-cut	74 (40)	13 (24)	61 (47)	.01
Avulsion	28 (15)	9 (17)	19 (14)	
Crush	83 (45)	32 (59)	51 (39)	
Dominant hand injured				
Yes	75 (40)	25 (46)	50 (38)	.39
No	110 (60)	29 (54)	81 (62)	
Amputation level				
Distal to PIP or IP joint	115 (62)	38 (70)	77 (59)	.19
Proximal to PIP or IP joint	70 (38)	16 (30)	54 (41)	
Long-term postoperative outcomes^c^				
MHQ score, median (IQR) [range]	81.1 (68.4-91.0) [20.0-100]	79.7 (63.1-93.1) [31.4-100]	82.0 (69.2-90.6) [20.0-100]	.33
DASH score, median (IQR) [range]	5.8 (1.7-12.1) [0-64.2]	5.8 (0.8-16.5) [0-54.5]	5.8 (1.7-11.7) [0-64.2]	.61
Grip strength of injured hand, mean (SD) [range], kg	30.2 (11.9) [0-60.7]	28.1 (11.9) [0-54.0]	31.1 (11.9) [4-60.7]	.13
Pinch strength of injured hand, median (IQR) [range], kg				
Lateral	7.7 (6.0-9.3) [0-25.0]	7.8 (5.1-9.3) [0-22.3]	7.7 (6.0-9.3) [1.0-25.0]	.71
2 Points	5.0 (3.3-7.0) [0-19.3]	5.5 (4.0-7.0) [0.5-17.0]	5.0 (3.3-6.5) [0-19.3]	.33
3 Points	6.0 (4.2-7.7) [0-26.3]	6.2 (4.5-7.8) [0-15.7]	5.8 (4.0-7.7) [0-26.3]	.60
9-Hole test score for injured hand, median (IQR) [range], s	23 (20-28) [10-134]	22 (20-27) [12-134]	23 (20-29) [10-87]	.47
Pain score for injured hand, median (IQR) [range]	5 (0-25) [0-90.0]	15 (0-29) [0-85.0]	5 (0-20) [0-90]	.08

Abbreviations: DASH, Disabilities of the Arm, Shoulder, and Hand; IP, interphalangeal; IQR, interquartile range; MHQ, Michigan Hand Outcomes Questionnaire; PIP, proximal interphalangeal.

^a^ Data are presented as number (percentage) of patients unless otherwise indicated.

^b^ Two-sided *P* values derived from the unpaired *t* test (Wilcoxon test for nonnormal data) are presented for continuous variables, and 1-sided *P* values derived from the unpaired χ^2^ test are presented for categorical variables.

^c^ Outcome data were measured at least 12 months after injury. Medians (IQRs) are provided for data with a nonnormal distribution; means (SDs) are provided for data that are approximately normally distributed.

### Statistical Analysis

All analyses were performed using R, version 3.4.4 and R Studio, version 1.1.442 (R Foundation for Statistical Computing). Two-sided *P* values derived from the unpaired *t* test (Wilcoxon test for nonnormal data) are presented for continuous variables, and 1-sided *P* values derived from the unpaired χ^2^ test are presented for categorical variables. *P* < .05 was considered to be statistically significant.

## Results

A total of 185 patients (mean [SD] age, 45 [16] years; 156 [84%] male) were studied. The median number of fingers amputated was 1 (range, 1-5), and most amputations were distal to the proximal interphalangeal joint (115 [62%]) and affected the nondominant hand (110 [60%]) (Table 3). Additional summary statistics for the full patient cohort are found in the article by Chung et al.^10^

Using T-RL estimation, we estimated that patients for whom hand dexterity is a clinical priority should preferentially undergo finger replantation (Table 2). Alternatively, we estimated that patients for whom hand-related QOL is most important should undergo revision amputation if they injure the dominant hand and replantation otherwise (Table 2). Treating all patients with replantation may minimize patient-reported long-term pain compared with revision amputation (Table 2). Also, in patients for whom hand strength is paramount, the results of our analysis indicated that factors such as the number of fingers amputated, presence of thumb amputation, and age should be considered when determining treatment. Specifically, although there was some variability in the estimated decision rules across the analyses, our results suggest that patients with a single-finger or thumb amputation should receive revision amputation, but those with other injury characteristics should receive replantation (Table 2). Sensitivity analyses with complete cases revealed estimates similar to those found in our primary analysis (Table 2). Using the full patient cohort with imputed missing data, however, we were able to detect tailoring variables for the dexterity and pain outcomes (sensitivity analysis 3) (Table 2). Additional discussion of sensitivity results is given in the eAppendix in the Supplement.

## Discussion

In this analysis, we identified and assessed specific, personalized decision rules to optimize hand outcomes after traumatic finger amputation, demonstrated the multilateral aspects of postsurgical outcomes, and established the feasibility of applying T-RL statistical methods in relevant clinical settings. Clinical practice guidelines that incorporate evidence-based medicine exist in all areas of medicine and improve patient care and lower health care costs.^25,26^ However, given the inherent heterogeneity among patients, flexible guidelines that are able to accommodate patient heterogeneity are needed.^27,28^

Using statistical learning, we estimated that, if the principal clinical objective is to maximize the patient’s hand strength, patients whose injury involves a single finger should be treated with revision amputation, and patients with other injury characteristics may be appropriately treated with replantation. This outcome may be most important for individuals employed in construction trades or childcare, for example. Our finding is consistent with other literature, including the study by Chung et al,^10^ that demonstrated improvement in strength for replantation for multiple finger amputations.^7,13^

For other patients, hand dexterity may be more important than hand strength, for example, in highly tactile jobs that require fine motor ability, such as those of a musician or hair stylist. If hand dexterity is of primary clinical importance, our results suggest that patients should preferentially undergo replantation. This finding of improved dexterity in the replantation cohort compared with the revision amputation cohort is consistent with the study by Chung et al,^10^ although few prior studies^29,30^ have focused on dexterity as a key functional outcome. Hand dexterity is achieved through the interfacing of complex biomechanics of the hand and neurocircuitry in the central and peripheral nervous system,^31^ which is clinically challenging to measure and interpret. However, such fine motor dexterity plays a significant role in activities of daily life^32^ and is essential to optimize in patients undergoing hand surgery.

Historically, most outcomes in medicine have focused on functional measures, for example, how much recovery has been achieved in terms of finger strength or what degree of rotation has been achieved after replantation. However, the hand surgery literature^7,11,33^ has suggested that patient-reported outcomes designed to capture hand function may be equally important in characterizing a patient’s recovery after surgery. In our analysis, we explored decision rules to estimate maximal patient-reported QOL. Our results suggest that, to maximize hand-related QOL, dominant hand injuries should be treated with revision amputation and nondominant hand injuries with replantation. This result is interesting for 2 reasons. First, although this finding has been identified by other investigators,^34^ it is counterintuitive and contrary to current practice, which prescribes treatment of replantation in the case of dominant hand injuries to maximize the utility and dexterity of the dominant hand. The literature reports cases of bilateral finger amputations in which amputated nondominant fingers were replanted to the dominant hand to maximize dexterity.^35^ Our analysis suggests that clinical intuition is not always consistent with evidence-based medicine, which further supports the need for and value of data-driven decision-making in hand surgery and other fields. Our analysis also suggests that there may be psychosocial repercussions associated with replantation that may not have been considered previously. We suspect that for finger replantations on the dominant hand the stiffness in the replanted finger(s) compared with the remaining uninjured fingers may impede the overall hand function when performing activities of daily living; however, the reason behind lower QOL in dominant hand finger replantations necessitates further investigation.

For some patients, an overall reduction in pain is of primary importance. When this is the case, we found that treatment of all patients undergoing finger amputation with replantation was associated with the greatest overall reduction in pain. This finding is consistent with previously published literature^6,13^ and likely occurs because during replantation the damaged nerve endings are trimmed and coapted together to encourage sensory return. However, this procedure secondarily decreases the probability of neuroma formation. In revision amputation, the nerve is no longer in continuity, increasing the chances of painful neuroma formation. Although pain after revision amputation or replantation has been discussed in several studies,^9,13^ its effects on overall QOL have not been thoroughly investigated in the traumatic finger amputee, especially given that nerve injury and pain lead to decreased activity levels, worse QOL, and higher depression scores.^36^

Prior studies^9,12,13,24^ have reported differences in outcomes that vary depending on the location of injury relative to the proximal interphalangeal or interphalangeal joint or the mechanism of injury. Our analysis did not identify either of those factors in any treatment rule, possibly because of a lack of statistical power in the sample size or that, by chance, these treatment interactions were not observed in the patient cohort in our study.

Of particular interest to the clinical community is the awareness that there is no one-size-fits-all approach to treating traumatic finger amputations, and to date, there is no treatment approach for traumatic finger amputation that unilaterally improves all aspects of hand function and QOL. Depending on which aspect of hand function or QOL is most important to the patient, a different treatment decision may be chosen based on the patient’s lifestyle and priorities, as well as their personal and injury characteristics. Although finger amputation is a traumatic injury that requires rapid intervention, a set of decision rules designed to incorporate patient preference by considering a patient’s unique, long-term functional needs can be useful for counseling patients on treatment choice.^7^

In addition, our study demonstrated the application of novel statistical learning techniques, which can provide additional value as a supplement to standard analysis methods.^37,38^ First, the principal goal of the T-RL method is to identify tailoring variables and their associated cut points such that, if the patient population were to follow this regimen, outcomes across the population may be optimized. This method is in contrast to standard methods, such as regression modeling, which does not share this primary goal. Second, the T-RL method combines semiparametric regression with flexible tree-based learning, meaning that fewer modeling assumptions are needed compared with classic regression modeling. Third, whereas propensity score modeling techniques can be applied within a regression analysis to minimize bias e, the T-RL method incorporates a purity measure that models the expected counterfactual mean outcome, thus providing a straightforward approach for addressing biases. Fourth, although we applied the T-RL method in a single-stage treatment setting, the premier value of the T-RL method is its ability to estimate decision rules across multiple treatment stages, reflecting the manner in which clinical care is provided.

### Limitations

This study has limitations. First, the retrospective, multisite nature of this study restricted baseline variables to those collected as part of standard, site-specific medical care at the time of injury; thus, information bias was likely. Second, we expect a potentially large degree of selection bias^39^ owing to the study design and enrollment^10^ or the analysis choices, which may mean that the sample was not representative of the population of interest. In this analysis, we included only patients for whom complete data were available, which we consider to be more prudent given that decision rules can be used for clinical care and several outcome variables had a large degree of missing data. Sensitivity analyses using the full patient cohort with imputed missing data identified similar tailoring variables for the estimated decision rules of hand strength and patient-reported function and, in contrast to the analyses with complete cases, identified age as a tailoring variable in the decision rules for dexterity and pain. Third, surgeon expertise was not controlled because it is difficult to measure. However, all participating sites are high-volume hand trauma centers with surgeons familiar with finger amputation management.

There are several assumptions that underlie our methods and analysis that should be noted. We assumed that data on all confounding variables were collected; however, given that the study did not collect data on patient-level comorbidities and smoking status or injury characteristics, such as duration of ischemia and amount of soft-tissue damage, which are also used in decisions that pertain to traumatic amputations,^7,13^ this assumption may not be fulfilled. We also assumed that patients with their unique set of baseline covariates could be assigned to either treatment group. However, treatment patterns differed between the United States and Asia^40^; which results in a low degree of overlap in the estimated propensity scores of patients with revision amputation and replantation, making causal methods more challenging.

## Conclusions

In this analysis, we described an evidence-based clinical decision support tool that may be able to assist surgeons in making personalized treatment decisions for patients with traumatic finger amputation. The findings suggest that there is no prescribed approach to treating traumatic finger amputations. We demonstrated the application of statistical learning techniques to supplement standard statistical analyses and to provide data-driven, evidence-based solutions for an important clinical condition.

## Notes

Supplement. **eAppendix.** Analysis Methods: Additional Detail **eTable.** Summary Statistics for Patient Demographics, Injury Characteristics, and Outcomes for Patients With No Missing Data (Complete Case Cohort) and the Full Patient Cohort With Imputed Missing Data
