# Supplementary material for: Assessment of Tree-Based Statistical Learning to Estimate Optimal Personalized Treatment Decision Rules for Traumatic Finger Amputations
Source: JAMA Netw Open. 2020 Feb 21;3(2):e1921626. doi: 10.1001/jamanetworkopen.2019.21626 (PMC7043191; doi:10.1001/jamanetworkopen.2019.21626)
Supplement: Supplement. — eAppendix. Analysis Methods: Additional Detail eTable. Summary Statistics for Patient Demographics, Injury Characteristics, and Outcomes for Patients With No Missing Data (Complete Case Cohort) and the Full Patient Cohort With Imputed Missing Data [file jamanetwopen-3-e1921626-s001.pdf]

Supplementary Online Content

Speth KA, Yoon AP, Wang L, Chung KC; FRANCHISE Group. Assessment of tree-based statistical learning to estimate optimal personalized treatment decision rules for traumatic finger amputations. *JAMA Netw Open*. 2020;3(2):e1921626. doi:10.1001/jamanetworkopen.2019.21626

eAppendix. Analysis Methods: Additional Detail

eTable. Summary Statistics for Patient Demographics, Injury Characteristics, and Outcomes for Patients With No Missing Data (Complete Case Cohort) and the Full Patient Cohort With Imputed Missing Data

This supplementary material has been provided by the authors to give readers additional information about their work.

© 2020 Speth KA et al. *JAMA Network Open.*

eAppendix. Analysis Methods: Additional Detail

Propensity estimation

For a multitude of reasons, a comparison of outcomes for patients treated with revision amputation versus replantation is not possible in the context of a randomized trial[1,2]. With observational data, however, although a causal interpretation is of course desired, a direct comparison of outcomes between treatment groups cannot be made. Confounding, which occurs when there are associations between one or more covariates and both the outcome of interest and the actual treatment assignment mechanism, can generate bias in the estimation when left uncorrected. This concern is frequently observed, for example, when the distributions of baseline covariates differ across treatment groups.

One statistical tool to remedy the issue of confounding in observational studies is the propensity score, i.e., the probability of treatment assignment conditional on baseline covariates. The importance of propensity score adjustment in the analysis of observational data is well documented[3–5]. Given the propensity score, the distribution of covariates will be similar when comparing across the observed treatment groups. We estimate the probability of receiving replantation using all covariates considered for a treatment decision rule and all potential confounders, i.e., the propensity score. A popular method of statistical analysis used when analyzing observational data is known as inverse probability of treatment weighting (IPW), which generates a synthetic sample of data (i.e., a pseudo-sample) using the inverse of the propensity score such that, in the pseudo-sample, baseline covariates are independent of treatment assignment and a direct comparison of the outcomes between treatments can be made.

We estimate propensity scores using the SuperLearner algorithm accessed through the SuperLearner package in R (version 2.0-24). SuperLearner is an algorithm that uses cross-validation to estimate best fit across multiple machine learning models and then combines these predictions, i.e., an "ensemble", using a weighted average based on the test data performance[6]. Prediction models available within the SuperLearner algorithm include generalized linear models via penalized maximum likelihood, random forest, ridge regression, generalized additive models, and others. SuperLearner has been shown to be asymptotically as accurate as any of the prediction methods used within the algorithm[6].

To generate propensity score estimations, we input into the SuperLearner algorithm with a dataset comprised of the actual treatment assignments (i.e., either revision amputation or replantation), the set of variables being considered as a treatment rule (refer to primary manuscript Table 1), and the set of all potential confounders. We run the SuperLearner using a binomial family with default hyperparameter settings under various assumptions, including the percentages in the test and training sets, and assign as the propensity score for our analyses the median of these estimates. Although we do not apply a pure IPW estimator in our T-RL algorithm, we assess covariate balance conditional on propensity estimates using the cobalt[7] package in R (version 3.6.1).

The mean and median propensity score estimates across all patients are 0.68 and 0.73, respectively, with a minimum of 0.04 and a maximum of 0.96. Although overlap of the propensity estimates based on the actual treatment received is low (refer to primary manuscript Figure 2A), covariate balance after propensity score adjustment is adequate for all variables except age, number of digits amputated, US site, race, and work-related injury (Figure 2B).

Tree-based reinforcement learning (T-RL)

One method that has been widely used to achieve a high level of prediction performance for continuous outcomes--and a concept upon which T-RL is based--is a regression tree. A regression tree is a nonparametric, supervised machine learning technique in which a sequence of binary partitions of the covariate space are made in order to achieve at each split the maximum purity of a continuous outcome[8,9], which is often consistent with minimizing the prediction error. The process of splitting nodes of the tree into binary partitions continue until the pre-specified depth of the tree is achieved or until the improvement made by continued partitioning of the covariate space falls below a pre-specified level. The results of a regression tree can be displayed in the form of a decision tree depicting each covariate split and summarizing the average estimated outcome for patients that fall within each leaf of the tree. Regression trees are implemented widely today due to their flexibility and interpretability.

There are two primary differences between a standard regression tree and T-RL[10]. First, the target of estimation is an *optimal treatment regimen* to maximize an outcome across a population, which is not directly observed, in contrast to a regression tree which estimates a continuous outcome on each leaf of the tree. Second, the purity measure used in T-RL differs from that used in a regression tree. Instead of

© 2020 Speth KA et al. *JAMA Network Open.*

using the sum of squared prediction errors, which is a commonly used purity measure for regression trees, T-RL must utilize a purity measure that reflects a difference in counterfactual outcomes, as is appropriate for a method that exists within the space of causal inference. This purity measure, which is evaluated at each possible split and selects the split that yields the maximum purity, just as in a regression tree, is the augmented inverse probability weighted (AIPW) estimator of the expected counterfactual mean[11] (1).

$$\max_{a_1, a_2 \in \mathcal{A}_j} \mathbb{P}_n \left[\sum_{a_j=1}^{K_j} \hat{\mu}_{j,a_j}^{AIPW}(\mathbf{H}_j) I\{g_{j,\omega,a_1,a_2}(\mathbf{H}_j) = a_j\} I(\mathbf{H}_j \in \Omega) \right] \quad (1)$$

This purity measure is the mechanism through which a series of binary partitions of the covariate space at each node of the tree, Omega, are made. It is doubly robust in the sense that the estimator will provide an unbiased estimate if either the propensity model or the conditional mean outcome model is correctly specified. Further details about the purity measure can be found in Tao et al.[10].

Although T-RL is designed to accommodate a sequence of successive treatment stages and to output an estimated, multi-stage decision rule, in this analysis we estimate a decision for only a single treatment stage. For a single stage treatment regimen, the T-RL requires input of a propensity model and a conditional mean model of the outcome. Estimation of the propensity score is described above. The conditional mean model of the outcome given treatment and covariate history, i.e, E(Y | A, H), is also unknown and is estimated using standard regression methods assuming a linear relationship of the outcome Y with the covariates H and treatment-covariate interactions HsubC + A*Hsub. A regression tree is then implemented using the T-RL AIPW purity measure to determine the sequence of binary partitions of the covariate space that will optimize the long-term counterfactual mean outcome at each split, as desired. We estimate treatment decision rules with a maximum depth of 2, given the hypothesis-generating nature of the method, a minimum number of 20 observations per leaf, and using a minimum percent increase of 5% required to permit a split of the covariate space to occur.

Sensitivity Analyses

Sensitivity Analysis 1 (Complete cases using only injury group as a possible treatment assignment variable, as per Chung et al.[12])

When using only a single covariate that represents the degree of severity of a traumatic amputation injury in a possible treatment assignment rule we found that, if the treatment goal is to maximize dexterity or hand-related quality of life, or to minimize pain, patients should preferentially receive replantation (refer to primary manuscript Table 3). If the treatment goal is to maximize long-term strength, patients with two or more digits amputated or with a single finger amputation (excluding thumb) distal to the PIP joint should receive replantation (refer to primary manuscript Table 3). If the patient has suffered a single thumb-only amputation or a single digit excluding thumb proximal to the PIP joint, however, he/she should undergo revision amputation (refer to primary manuscript Table 3). Chung et al.[12] also reports better hand-related quality-of-life overall for replantation patients compared with those receiving revision amputation. The authors also recommend replantation of single digit amputations (excluding thumb) distal to the PIP joint, which coincides with our estimated decision rule for the composite outcome of strength. However, for the outcomes of dexterity, which was based on the 9-hole timed test, and 3-point pinch strength, Chung et al.[12] identified better outcomes for replantation in subgroups with multiple digit amputations. There are several reasons for these discrepancies. First, we do not account for correlation of outcomes across study center, as was performed in Chung et al.[12], although new statistical methods to accommodate this correlation are forthcoming by our research group. We do, however, account for study center as a potential confounding variable. Secondly, with the exception of a single digit (excluding thumb) distal to the PIP joint, there are relatively few complete case observations within each subgroup (range: 6 to 25). Furthermore, although the subgroup analyses performed by Chung et al.[12] do include a center-level random intercept, the random intercept models do not include an adjustment for propensity score. Finally, our outcomes represent a composite of several measures. Strength, for example, incorporates grip strength, key pinch strength, 2-point pinch strength,

and 3-point pinch strength. Similarly, hand-related quality-of-life outcome incorporates both MHQ and DASH measures. The associations identified by Chung et al.[12] refer to specific outcome measures, so it is possible that the effect of a composite outcome fails to identify the same associations.

Sensitivity Analysis 2 (Complete cases using injury characteristics, but excluding age, as stand-alone variables in a possible treatment assignment variable)

In our complete case analysis using injury characteristics as individual variables that can be included in an estimated decision rule, we identify the same estimated treatment rules for the composite outcomes of dexterity, hand-related quality-of-life, and pain (refer to primary manuscript Table 3), which makes sense given that age is not identified in any of the estimated treatment rules for these outcomes. For strength, we estimate that patients who sustain a single digit amputation of the thumb should receive revision amputation and all other patients should receive replantation in order to maximize long-term strength across the patient population (refer to primary manuscript Table 3). Although this finding is contradictory to the traditional teaching that aims to preserve the length and function of the thumb given its integral role in hand function, it is possible that the principal reason for this treatment norm is related to factors other than hand strength, e.g., grasp. If the clinical objective were to improve hand strength overall, it has been shown that a thumb improves strength only marginally over that of a full hand grip[13].

Sensitivity Analysis 3 (Full patient cohort with missing data imputed using random forest)

Fifty-five percent (55%) of patients have complete covariate and outcome data. For the remaining 153 patients we impute missing data fields using random forest imputation[14]. Summary statistics of demographics, injury characteristics, and outcomes for the complete case and the full imputed datasets are presented in eTable 1. The patient in the primary analysis (reported in the main text) is slightly younger and is more likely to have been treated with replantation than that found in the full patient cohort. Due to the manner in which the outcomes were collected, this resulted in disproportionately more patients treated in the United States being excluded from the analysis. Otherwise, however, baseline demographics and injury characteristics between the complete cases and the full patient cohort are similar.

Using a full patient cohort with imputed missing data, the estimated treatment rule to maximize hand-related quality of life is the same as the one we identified in the main analysis, i.e., that patients with dominant hand injuries should be treated with revision amputation and replantation for non-dominant hand injuries (refer to primary manuscript Table 3). This could be because the degree of missing data for the MHQ and DASH questionnaires was less than 3% and the data obtained from the complete cases was representative of the full patient cohort.

The estimated decision rule for our composite outcome of strength also identified number of digits amputated and age as factors determining the optimal decision rule; however, the cut points for each variable differed (refer to primary manuscript Table 3). The imputed dataset suggests that patients with four or more digits amputated or who are 74 years or older should receive replantation. Otherwise, revision amputation is recommended to maximize long-term hand strength. Because the strength outcomes have a low degree of missingness, also less than 3% overall, we may be observing bias that is seen in the complete cases compared with the full patient cohort. Given the low degree of missingness in the strength outcomes, we might suggest that the results presented here are actually more representative of our patient population than that presented in the primary analysis.

Whereas our main analysis independently recommends all patients be treated with replantation in order to optimize long-term dexterity and pain, the results of this sensitivity analysis identifies age as a tailoring in both treatment assignment rules (refer to primary manuscript Table 3). For both the dexterity and pain outcomes, we identify age as a tailoring variable that can be used to determine treatment to maximize long-term dexterity or to minimize pain following traumatic digit amputation, perhaps suggesting more precision of the statistical learning method given additional data. Given the high degree of missingness (30%) in the dexterity and pain outcomes, however, and the single imputation performed to impute these data, we note this as a finding for additional investigation.

eReferences

1. Chung KC, Alderman AK. Replantation of the upper extremity: Indications and outcomes. *Journal of the American Society for Surgery of the Hand*. 2002;2(2):78-94.
2. Sebastin SJ, Chung KC. A systematic review of the outcomes of replantation of distal digital amputation. *Plast Reconstr Surg*. 2011;128(3):723-737.
3. Miguel A Hernán JMR. *Causal Inference*. Boca Raton: Chapman & Hall/CRC, forthcoming; 2019.
4. Chakraborty B, Moodie EEM. *Statistical Methods for Dynamic Treatment Regimes: Reinforcement Learning, Causal Inference, and Personalized Medicine*. Springer New York; 2013.
5. Austin PC. An Introduction to Propensity Score Methods for Reducing the Effects of Confounding in Observational Studies. *Multivariate Behav Res*. 2011;46(3):399-424.
6. Polley EC, van der Laan MJ. Super Learner In Prediction: Working Paper 266. *UC Berkeley Division of Biostatistics Working Paper Series*. 2010. https://biostats.bepress.com/ucbbiostat/paper266.
7. Greifer N. *Cobalt: Covariate Balance Tables and Plots*.; 2019.
8. Loh W-Y. Classification and regression trees. *WIREs Data Mining Knowl Discov*. 2011;1(1):14-23.
9. Gareth James, Daniela Witten, Trevor Hastie, Robert Tibshirani. *An Introduction to Statistical Learning with Applications in R*. Springer; 2015.
10. Tao Y, Wang L, Almirall D. Tree-based reinforcement learning for estimating optimal dynamic treatment regimes. *Ann Appl Stat*. 2018;12(3):1914-1938.
11. Tao Y, Wang L. Adaptive contrast weighted learning for multi-stage multi-treatment decision-making. *Biometrics*. 2017;73(1):145-155.
12. Chung KC, Yoon AP, Malay S, et al. Patient-Reported and Functional Outcomes After Revision Amputation and Replantation of Digit Amputations: The FRANCHISE Multicenter International Retrospective Cohort Study. *JAMA Surg*. April 2019. doi:10.1001/jamasurg.2019.0418
13. Cha SM, Shin HD, Kim KC, Park JW. Comparison of grip strength among 6 grip methods. *J Hand Surg Am*. 2014;39(11):2277-2284.
14. Tang F, Ishwaran H. Random Forest Missing Data Algorithms. *Stat Anal Data Min*. 2017;10(6):363-377.

© 2020 Speth KA et al. *JAMA Network Open.*

eTable. Summary Statistics for Patient Demographics, Injury Characteristics, and Outcomes for Patients With No Missing Data (Complete Case Cohort) and the Full Patient Cohort With Imputed Missing Data

		**Full imputed dataset**			**Complete case cohort**		
	**N (%)**	**Overall (n=338)**	**Revision amputation (n=161)**	**Replantation (n=177)**	**Overall (n=185)**	**Revision amputation (n=54)**	**Replantation (n=131)**
**Demographic Characteristics**							
Age, mean (SD) [range]	338 (100)	48 (16.5) [18-83]	51 (17.3) [20-83]	45 (15.2) [18-80]	45 (16.0) [18-82]	49 (17.9) [21-82]	44 (15.0) [18-80]
Gender: Male, No. (%)	338 (100)	287 (84.9)	134 (83.2)	153 (86.4)	156 (84.3)	45 (83.3)	111 (84.7)
Treatment location, No. (%)	338 (100)						
United States		136 (40.2)	111 (68.9)	25 (14.1)	27 (14.6)	15 (27.8)	12 (9.2)
Asia		202 (59.8)	50 (31.1)	152 (85.9)	158 (85.4)	39 (72.2)	119 (90.8)
**Baseline Injury characteristics**							
Number of digits amputated, mean (SD) [range]	338 (100)	1.7 (1.1) [1-8]	1.5 (0.9) [1-6]	1.8 (1.2) [1-8]	1.6 (1.0) [1-5]	1.3 (0.8) [1-4]	1.7 (1.0) [1-5]
Thumb amputated, No. (%)	338 (100)	85 (25.1)	32 (19.9)	53 (29.9)	47 (25.4)	10 (18.5)	37 (28.2)
Mechanism of injury, No. (%)	299 (88)						
Clean cut		136 (45.5)	55 (43.3)	81 (47.1)	74 (40.0)	13 (24.1)	61 (46.6)
Avulsion		44 (14.7)	16 (12.6)	28 (16.3)	28 (15.1)	9 (16.7)	19 (14.5)
Crush		119 (39.8)	56 (44.1)	63 (36.6)	83 (44.9)	32 (59.3)	51 (38.9)
Dominant hand injured, No. (%)	338 (100)	138 (40.8)	64 (39.8)	74 (41.8)	75 (40.5)	25 (46.3)	50 (38.2)
Amputation level, No. (%)	338 (100)						
Distal to PIP/IP		215 (63.6)	115 (71.4)	100 (56.5)	115 (62.2)	38 (70.4)	77 (58.8)
Proximal to PIP/IP		123 (36.4)	46 (28.6)	77 (43.5)	70 (37.8)	16 (29.6)	54 (41.2)
**Long-term Postoperative Outcomes**							
MHQ, mean (SD) [range]	328 (97)	76.9 (18.0) [18.7-100]	77.1 (18.4) [23-100]	76.8 (17.6) [18.7-100]	77.8 (17.0) [20.0-100]	75.2 (18.8) [31.4-100]	78.9 (16.1) [20.0-100]
DASH, mean (SD) [range]	333 (99)	12.4 (15.3) [0-95.8]	13.6 (15.6) [0-70.5]	11.3 (14.9) [0-95.8]	9.7 (11.7) [0-64.2]	11.6 (14.3) [0-54.5]	8.9 (10.5) [0-64.2]
Grip strength, kg (injured), mean (SD) [range]	333 (99)	31.8 (13.8) [0-90]	31.4 (13.7) [0-75.3]	32.1 (13.8) [4-90]	30.2 (11.9) [0-60.7]	28.1 (11.9) [0-54.0]	31.1 (11.9) [4-60.7]
Lateral pinch strength, kg (injured), mean (SD) [range]	332 (98)	8.7 (3.9) [0-27]	8.9 (3.7) [0-22.3]	8.5 (4.1) [0-27]	7.7 (3.1) [0-25.0]	7.7 (3.7) [0-22.3]	7.7 (2.8) [1.0-25.0]

© 2020 Speth KA et al. *JAMA Network Open.*

2-point pinch strength, kg (injured), mean (SD) [range]	331 (98)	5.8 (3.3) [0-23.3]	6.1 (3.0) [0-19.3]	5.6 (3.5) [0-23.3]	5.2 (2.8) [0-19.3]	5.5 (3.0) [0.5-17.0]	5.0 (2.7) [0-19.3]
3-point pinch strength, kg (injured), mean (SD) [range]	326 (96)	6.7 (3.8) [0-30]	6.9 (3.2) [0-21.3]	6.6 (4.3) [0-30.0]	6.0 (3.2) [0-26.3]	6.1 (3.2) [0-15.7]	6.0 (3.3) [0-26.3]
9-hole test, seconds (injured), mean (SD) [range]	238 (70)	27.1 (13.2) [10-134]	28.4 (18.0) [11-134]	26.5 (10.4) [10-87]	26.1 (13.4) [10-134]	27.4 (19.3) [12-134]	25.6 (10.1) [10-87]
Pain (injured), mean (SD) [range]	231 (68)	16.7 (21.1) [0-100]	20.3 (22.0) [0-85]	15.3 (20.6) [0-100]	14.7 (18.9) [0-90.0]	18.8 (21.1) [0-85.0]	13.0 (17.7) [0-90]

SD = standard deviation; No. = number; PIP = Proximal interphalangeal joint; IP = Thumb interphalangeal joint; MHQ = Michigan Hand Outcomes Questionnaire; DASH = Disabilities of the Arm, Shoulder, and Hand; kg = kilograms.

© 2020 Speth KA et al. *JAMA Network Open.*
